# Cytogenetic characterisation and chromosomal mapping of microsatellite and telomeric repeats in two gecko species (Reptilia, Gekkonidae) from Thailand

**DOI:** 10.3897/CompCytogen.v15i1.58208

**Published:** 2021-02-02

**Authors:** Weera Thongnetr, Surachest Aiumsumang, Rodjarin Kongkaew, Alongklod Tanomtong, Chatmongkon Suwannapoom, Sumalee Phimphan

**Affiliations:** 1 Walai Rukhavej Botanical Research institute, Mahasarakham University, Kantharawichai, Maha Sarakham, Thailand Mahasarakham University Maha Sarakham Thailand; 2 Biology program, Faculty of Science and Technology, Phetchabun Rajabhat University, Phetchabun, 67000, Thailand Phetchabun Rajabhat University Phetchabun Thailand; 3 Program of Biology, Faculty of Science, Khon Kaen University, Muang, Khon Kaen, 40002, Thailand Khon Kaen University Khon Kaen Thailand; 4 Department of Fishery, School of Agriculture and Natural Resources, University of Phayao, Muang, Phayao, 56000, Thailand University of Phayao Phayao Thailand

**Keywords:** Ag-NOR, *Cyrtodactylus doisuthep*, *Cyrtodactylus jarujini*, FISH microsatellite, karyotype

## Abstract

Studies of chromosomes of *Cyrtodactylus jarujini* Ulber, 1993 and *C. doisuthep* Kunya et al., 2014 to compare microsatellite and TTAGGG sequences by classical and molecular techniques were conducted in Thailand. Karyological typing from a conventional staining technique of *C. jarujini* and *C. doisuthep* showed diploid chromosome numbers of 40 and 34 while the Fundamental Numbers (NF) were 56 in both species. In addition, we created the chromosome formula of the chromosomes of *C. jarujini* showing that 2n (40) = L^sm^_1_ + L^sm^_2_ + L^t^_3_ + M^m^_1_ + M^t^_4_ + S^m^_2_ + S^a^_2_ + S^t^_5_ while that of *C. doisuthep* was 2n (34) = L^sm^_3_ + L^m^_2_ + L^t^_3_ + M^m^_1_ + M^t^_2_ + S^m^_4_ + S^a^_1_ + S^t^_1_. Ag-NOR staining revealed NOR-bearing chromosomes in chromosome pairs 13 and 14 in *C. jarujini*, and in chromosome pairs 9 and 13 in *C. doisuthep*. This molecular study used the FISH technique, as well as microsatellite probes including (A)_20_, (TA)_15_, (CGG)_10_, (CGG)_10_, (GAA)_10_, (TA)_15_ and TTAGGG repeats. The signals showed that the different patterns in each chromosome of the Gekkonids depended on probe types. TTAGGG repeats showed high distribution on centromere and telomere regions, while (A)_20_, (TA)_15_, (CGG)_10_, (CGG)_10_, (GAA)_10_ and (TA)_15_ bearing dispersed over the whole genomes including chromosomes and some had strong signals on only a pair of homologous chromosomes. These results suggest that the genetic linkages have been highly differentiated between the two species.

## Introduction

**Figure 1.**
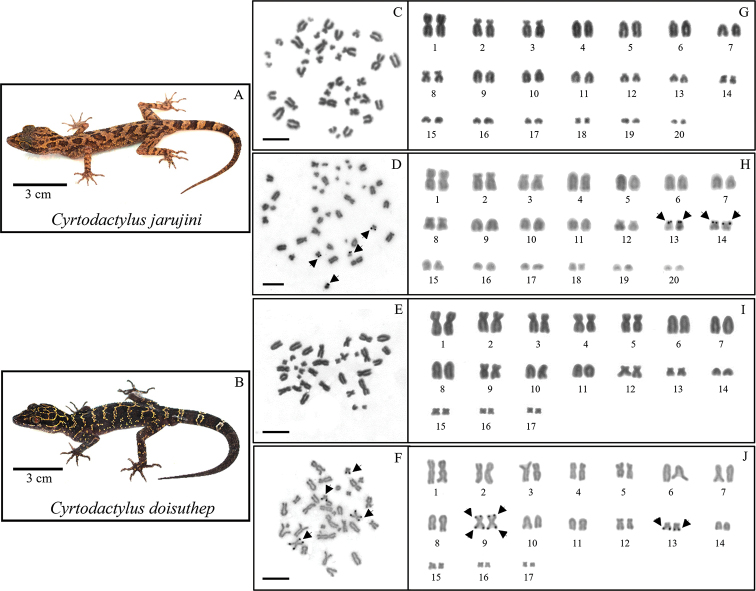
The *C. jarujini* specimen (**A**), metaphase chromosome plate and karyotypes (**A–G**) by conventional technique, (**D–H**) by Ag-NOR banding technique. The *C. doisuthep* specimen (**B**), metaphase chromosome plate and karyotypes (**E–I**) by conventional technique, (**F–J**) by Ag-NOR banding technique. Arrows indicated Ag-NORs regions. Scale Bar: 5 µm.

Bent-toed geckos (genus *Cyrtodactylus* Gray, 1827) in Thailand have been classified into approximately 24 species (Chuaynkern and Chuaynkern 2012). *Cyrtodactylus jarujini* ranges from Nong kai, Bueng Kan and Nakhon Phanom Provinces, Thailand. More recently, Sumontha et al. (2008), found it in two caves on two sandstone hills, Phu Sing and Phu Thok, where it remained by day on the walls and crevices and emerged from the caves at night. Both in Phu Sing and Phu Thok, syntropy was found with the cave-dwelling agamid *Mantheyus phuwuanensis* (Manthey and Nabhitabhata 1991). It has also been recorded from central and northern Laos (Stuart 1999), but the exact identity of the Lao populations has to be re-evaluated (Fig. 1A). In contrast *C. doisuthep* is known only from Doisuthep in the Doi Suthep-Pui Range, Mueang District, Chiang Mai Province, northern Thailand (Fig. 1B).

Only 13% of gekkonid species have been karyotyped (Olmo and Signorino 2005) and were studied with conventional cytogenetic methods, including routine staining, as well as R-, NOR- and C-banding (Moritz 1983; Olmo and Signorino 2005; Shibaike et al. 2009). However, a small number of species were studied by molecular cytogenetic techniques (Kawai et al. 2009). The diploid number amongst gekkonid lizards ranges from 2n = 28 to 46 with most of the karyotypes composed of 28–46 chromosomes (Gorman 1973; Olmo 1986; Schmid et al. 1994). There are five karyotyped *Cyrtodactylus* species: *C. consobrinus* 2n = 48, NF = 50, *C. pubisulcus* 2n = 42, NF = 44 (Ota et al. 1992); *C. interdigitalis* 2n = 42, NF = 52 and *C. kunyai* 2n = 40, NF = 52 (Thongnetr et al. 2019a); *C. saiyok* 2n = 42, NF = 42 (Thongnetr et al. 2019b). The typical karyotype consists of a gradual series of telocentric chromosomes (sometimes with a few metacentric) and there is no distinction between macro- and microchromosomes, the centromere often being subterminal (Gorman 1973). Karyotype evolution within the group is accompanied by fissions and fusions and pericentric inversions (Gorman 1973; Olmo and Signorino 2005). This information on chromosomes is considered important along with other information for identification of the species (Campiranont 2003), especially the identification of related species, because of similarity of shape, appearance and other phenotypic expressions that are presumed to be associated with the genotype. Information from sequences of DNA allows us to understand the creation of a phylogenetic tree (dendrogram), because these characteristics often have a particular pattern. Information on chromosomes can be used to identify the phylogenetic relationship between species and population of animals (Lauhajinda and Taksintum 2006). Therefore, it is necessary to study the karyology of this group. In addition, geckos could be affected by the actions of humans in their use of household objects and agricultural chemicals. Thus, the gecko is one of the important groups of animals that can serve as a model for studying the environmental impact from human actions in the future.

## Material and methods

The samples of *C. jarujini* and *C. doisuthep* were collected from the Phu Wua, Ban Phaeng District, Nakhonphanom Province and Doi Suthep-Pui Range, Mueang District, Chiang Mai Province, Thailand, (permission from an ethical committee ID U1-04498-2559). Chromosomes were directly prepared *in vivo* (Ota et al. 1990) by 0.1% colchicine were injected into the animals’ intramuscular and abdominal cavity and left for 8–10 hours. Bone marrow, liver and testis (male) were cut into small pieces and then mixed with 0.075 M potassium chloride (KCl). After discarding all large cell pieces, 15 ml of cell suspension was transferred to a centrifuge tube and incubated 30–40 minutes, then centrifuged at 3,000 rpm for 8 minutes. Cells were fixed in fresh cool fixative of methanol:glacial acetic acid (3:1) and gradually made up to 8 ml before centrifuging again at 3,000 rpm for 8 minutes, whereupon the supernatant was discarded. Fixation was repeated until the supernatant was clear and the pellet was mixed with 1 ml fixative. Using conventional Giemsa staining, a drop of the mixture was added to a clean and cold slide by micropipette followed by the air-dry technique. The slide was conventionally stained with 20% Giemsa solution for 30 minutes (Patawang et al. 2014). Then, the slides were rinsed thoroughly with running tap water to remove excess stain. Afterwards, the slides were allowed to air-dry at room temperature. Ag-NOR banding was analysed following the method of Howell and Black (1980). Two drops each of 50% silver nitrate and 2% gelatine solutions were added to slides, respectively. Then, they were sealed with cover glasses and incubated at 60 °C for 5–10 minutes. Afterwards, they were then soaked in distilled water until the cover glasses were separated. Finally, the slides were allowed to air-dry at room temperature and observed under microscope. Metaphase figures were analysed according to the chromosome classification of Chaiyasut (1989) and Turpin and Lejeune (1965). Chromosomes were classified as metacentric (m), submetacentric (sm), acrocentric (a) and telocentric (t). The Fundamental Number (NF: number of chromosome arms) is obtained by assigning a value of two to metacentric, submetacentric and acrocentric chromosomes and one to acrocentric chromosomes. The use of microsatellite probes described by Kubat et al. (2008) was followed here with slight modifications. These sequences were directly labelled with Cy3 at the 5´-terminal during synthesis by Sigma (St. Louis, MO, USA). Fluorescence *In Situ* Hybridization (FISH) was performed under highly stringent conditions on mitotic chromosome spreads (Pinkel et al. 1986). After denaturation of chromosomal DNA in 70% formamide/ 2×SSC at 70 °C, spreads were incubated in 2×SSC for 4 min at 70 °C. The hybridization mixture (2.5 ng/μL probes, 2 μg/μL salmon sperm DNA, 50% deionized formamide, 10% dextran sulphate) was dropped on the slides, and the hybridization was performed overnight at 37 °C in a moist chamber containing 2×SSC. The post hybridization wash was carried out with 1×SSC for 5 min at 65 °C. A final wash was performed at room temperature in 4×SSCT for 5 min. Finally, the chromosomes were counterstained with DAPI (1.2 μg/mL), mounted in antifading solution (Vector, Burlingame, CA, USA), and analyzed in fluorescence microscope Nikon ECLIPSE.

## Results

### The diploid chromosome number and fundamental number

The diploid numbers in *C. jarujini* and *C. doisuthep*, were 40 and 34, respectively (Fig. 1C, E), whereas NF was 56 in both species (Fig. 1G, I). The type chromosomes of metacentric, submetacentric, acrocentric and telocentric were 8-4-4-24 and 14-6-2-12. There are no sex-related chromosomal heteromorphisms in the two species here studied.

### The karyological characteristics

The karyotype of *C. jarujini* consists of two large metacentric, four large submetacentric, six large telocentric, two medium metacentric, eight medium telocentric, four small metacentric, four small acrocentric and ten small telocentric chromosomes. The karyotype formula for *C. jarujini* is as follows: 2n (40) = L^m^_2_ + L^sm^_4_ + L^t^_6_ + M^m^_2_ + M^t^_8_ + S^m^_4_ + S^a^_4_ + S^t^_10_ or 2n (40) = 8m + 4sm + 4a + 24t. The karyotype of *C. doisuthep* comprises four large metacentric, six large submetacentric, six large telocentric, two medium metacentric, four medium telocentric, eight small metacentric, two small acrocentric and two small telocentric chromosomes. The karyotype formula for *C. doisuthep* is as follows: 2n (34) = L^m^_4_ + L^sm^_6_ + L^t^_6_ + M^m^_2_ +M^t^_4_ + S^m^_8_ + S^a^_2_ + S^t^_2_ or 2n (34) = 14m + 6sm + 2a + 12t.

### Ag-NOR banding

This technique highlighted active NORs on pairs 13 and 14 of *C. jarujini* (Fig. 1D, H) and pairs 9 and 13 of *C. doisuthep* (Fig. 1F, J).

### Microsatellite pattern

**Figure 2.**
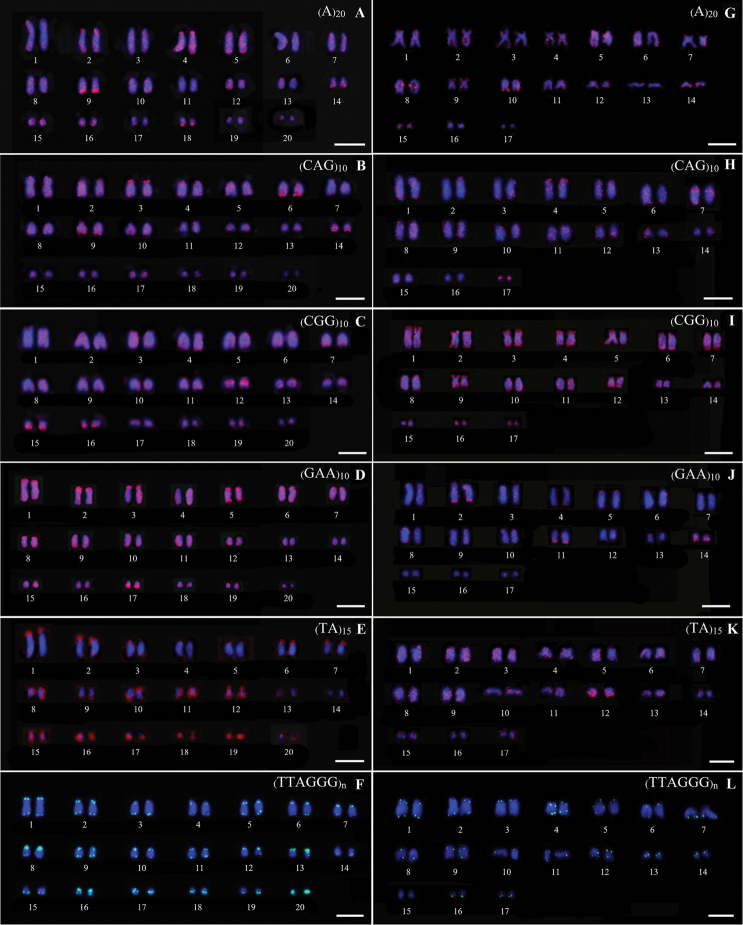
Karyotypes of two geckos presenting the patterns of microsatellite (A)_20_, (CAG)_10_, (CGG)_10_, (GAA)_10_, (TA)_15_ and TTAGGG; *C. jarujini* (**A–F**), *C. doisuthep* (**G–L**). Scale Bars: 10 µm.

Microsatellites (A)_20_, (TA)_15_, (CAG)_10_, (CGG)_10_, (GAA)_10_ and (TA)_15_ abundantly distributed in some chromosomes, usually in telomeric regions of both species studied. FISH with the telomeric probe TTAGGG revealed hybridization signals on each telomere of all chromosomes (Fig. 2).

## Discussion

### Karyological data of the genus Cyrtodactylus

The species in the *Cyrtodactylus* exhibited a variable chromosome number, ranging from 34 to 42, however, the most frequent numbers were 40 and 42. The present study showed that the chromosome numbers of *C. jarujini* and *C. doisuthep* were 40 and 34, respectively. The fundamental number was 56 in both species. These results showed difference and accordance with others *Cyrtodactylus* that have been reported (Table 1). The karyological characteristics of *C. jarujini* and *C. doisuthep* obtained in the present study are the first report of chromosome sizes and the chromosome types in these species. In different species of *Cyrtodactylus*, different karyological characteristics can be found. However, overall, of these karyotypes of *C. jarujini* and *C. doisuthep* resemble those of other *Cyrtodactylus* species and other gekkonids, which comprised many mono-armed (telocentric) and few bi-armed chromosomes (meta- or submetacentric). For those gekkonid chromosomes which have been reported previously, most species showed that the karyotype comprises of many mono-armed chromosomes and few bi-armed chromosomes. The present results of *C. jarujini* and *C. doisuthep* agreed with the chromosomal evolution line hypothesis within the gekkonid group (Trifonov et al. 2011). The karyotype of *C. jarujini* and *C. doisuthep* showed the gradient of most telocentrics, while comprising of a few bi-armed chromosomes. These features conform to the hypothesis of re-arrangement from ancestral karyotype by Robertsonian fissions, fusions or pericentric inversions (Gorman 1973; King 1987).

**Table 1.** Karyotype reviews in the genera Cyrtodactylus, Gekko Laurenti, 1768 and Hemidactylus Goldfuss, 1820 (Gekkonidae, Squamata).

**Species**	**2n**	**NF**	**Karyotype formula**	**NORs**	**Location**	**Reference**
*Cyrtodactylus consobrinus* (Peters, 1871)	48	50	2bi-arm+46t	–	Malaysia	Ota et al. (1992)
*C. doisuthep* Kunya et al., 2014	**34**	**56**	**14m+6sm+2a+12t**	**P9, 13**	**Thailand**	**Present study**
*C. interdigitalis* Ulber, 1993	42	52	4m+2sm+4a+32t	P12	Thailand	Thongnetr et al. (2019a)
*C. jarujini* Ulber, 1993	**40**	**56**	**8m+4sm+4a+24t**	**P13, 14**	**Thailand**	**Present study**
*C. kunyai* Pauwels et al., 2014	40	52	8m+4sm+6a+22t	P12	Thailand	Thongnetr et al. (2019a)
*C. pubisulcus* Inger, 1958	42	44	2bi-arm+40t	–	Malaysia	Ota et al. (1992)
*C. saiyok* Panitvong, 2014	42	42	42t	P15	Thailand	Thongnetr et al. (2019b)
*Gekko chinensis* Gray 1842	40	46	6bi-armed+34uni-armed	–	China	Lau et al. (1997)
*G. gecko* (Linnaeus, 1758)	38	50	12bi-armed+26uni-armed	–	–	Cohen et al. (1967)
	38	50	Lm2+Lsm4+Lt4+Mt6+Sm4+Sa2+St16	P4	Thailand	Patawang et al. (2014)
*G. hokouensis* Pope, 1928	38	56	4m+6sm+20t+8bi-armed	P(L)19	China	Chen et al. (1986)
*G. monarchus* (Schlegel, 1836)	38	46	–	–	Malaysia	Ota et al. (1990)
*G. petricolus* Taylor, 1962	38	54	–	–	–	Ota (1989)
*G. shibatai* Toda et al., 2008	38	58	4m+8sm+18t+8bi-armed	P(L)19	Japan	Shibaike et al. (2009)
*G. tawaensis* Okada, 1956	38	58	4m+8sm+18t+8bi-armed	P(L)19	Japan	Shibaike et al. (2009)
*G. taylori* Grossmann et Ulber, 1990	42	–	–	–	Thailand	Ota and Nabhitabhata (1991)
*G. vertebralis* Toda et al., 2008	38	62	4m+14sm+14t+6bi-armed	P(L)19	Japan	Shibaike et al. (2009)
*Hemidactylus brookii* Gray, 1854	40	44	4bi-armed+36t	–	–	Bhatnagar (1962)
*H. flaviviridis* Rüppell, 1835	40	60	20bi-armed+20t	–	–	Asana and Mahabale (1941)
	46	46	46t	–	–	Makino and Momma (1949)
	40	52	12bi-armed+28t	–	–	Branch (1980)
*H. frenatus* Schlegel, 1836	46	46	46t	–	–	Makino and Momma (1949)
	40	54	14bi-armed+26t	P3	–	King (1978)
	40	46	6bi-armed+34t	–	–	Darevsky et al. (1984)
*H. mabouia* (Moreau de Jonnès, 1818)	42	56	14bi-armed+28t	–	–	Becak et al. (1972)
	42	54	12bi-armed+30t	–	–	McBee et al. (1987)

**Remarks**: 2n = diploid chromosome number, NORs = nucleolus organiser regions, SCR = subcentromeric regions, NF = fundamental number (number of chromosome arms), bi-arm = bi-armed chromosome, m = metacentric, sm = submetacentric, a = acrocentric, t = telocentric chromosome, L = large, S = small, P = chromosome pair and – = not available.

### Active NOR sites

Nucleolus organiser regions (NORs) are chromosome sites which contain the 18S and 28S ribosomal RNA genes. If these regions were active during the interphase prior to mitosis, they can be detected by silver nitrate staining (Howell and Black 1980). In the present study, the chromosome markers of both *Cyrtodactylus* are determined by using the Ag-NOR banding technique as shown in Table 1. *C. jarujini* had the acrocentric chromosome pair 13 and metacentric chromosome pair 14, which were the NOR-bearing chromosome. Pair 13 NORs were located on the short arm near the telomere (telomeric NOR) and the pair 14 NORs were located on the short arm near the centromere (centromeric NOR). *C. doisuthep* had the metacentric, two chromosome pair 9 and pair 13 which were the NOR-bearing chromosomes. Pair 9 NORs were located on the arm near the telomere (telomeric NOR) on both sides and the pair 13 NORs were located on the arm near the telomere (telomeric NOR).

The NORs in both species of genus *Cyrtodactylus* exhibited at the telomeric region on the long arm and short arm and are similar to the previous reports of the gekkonids for the Gekkonidae family by King (1978) and Moritz and King (1985). The NORs of *Dixonius siamensis* (Boulenger, 1898), *G. gecko*, *G. hokouensis*, *G. shibatai*, *G. tawaensis*, *G. vertebralis*, *H. frenatus* and *H. platyurus* were found at all regions on the short arm and that agrees with those previous reported (Asana and Mahabale 1941; Makino and Momma 1949; Bhatnagar 1962; Cohen et al. 1967; Becak et al. 1972; King 1978; Branch 1980; Darevsky et al. 1984; Chen et al. 1986; McBee et al. 1987; Ota 1989; Ota et al. 1990; Ota and Nabhitabhata 1991; Lau et al. 1997; Ota et al. 2001; Shibaike et al. 2009; Patawang et al. 2014; Trifonov et al. 2011; Trifonov et al. 2015).

### Microsatellite pattern

**Figure 3.**
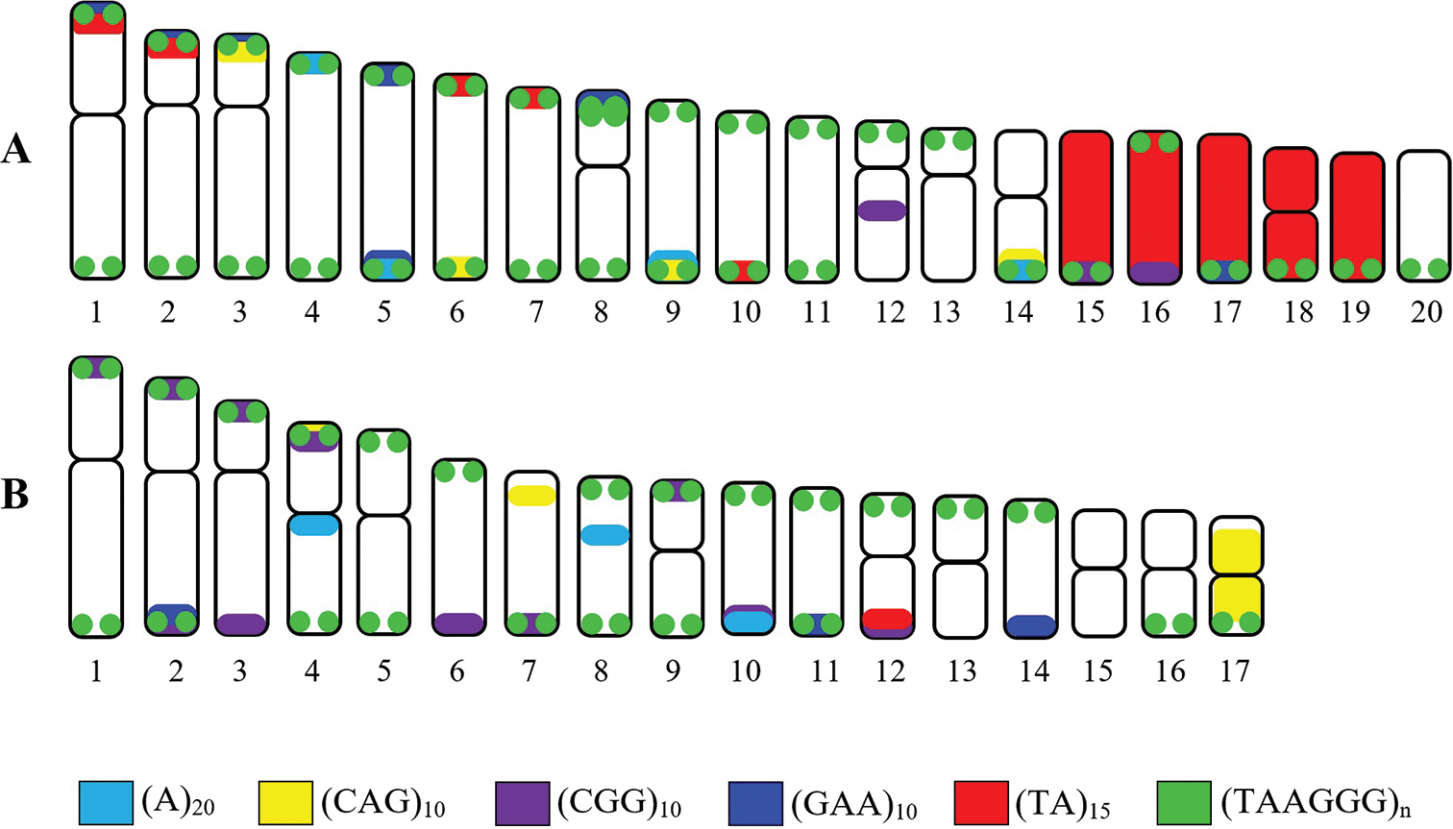
Idiograms represent the (A)_20_, (CAG)_10_, (CGG)_10_, (GAA)_10_, (TA)_15_ and TTAGGG mapping on the chromosomes of *C. jarujini* (**A**) and *C. doisuthep* (**B**).

Microsatellites or simple sequence repeats (SSRs) are oligonucleotides of 1–6 base pairs in length, forming excessive tandem repeats of usually 4 to 40 units (Tautz and Renz 1984; Ellegren 2004; Chistiakov et al. 2006). They show abundant distribution throughout eukaryotic genomes, being dispersed or clustered both in euchromatin or heterochromatin. They are highly polymorphic regarding copy number variations (Ellegren 2004). In our present study both species exhibited the same general hybridisation pattern for some applied probes with the motif TAAGGG repeat showing abundance at the telomeric ends of all chromosomes (Fig. 3), corroborating findings from other gekko groups studied to date (Srikulnath 2015). Otherwise, the dinucleotides (A)_20_, (CAG)_10_, (CGG)_10_, (GAA)_10_ and (TA)_15_ accumulated exclusively in telomeric and subtelomeric chromosomal regions. However, the results clearly indicate that the microsatellite repeats are in high copy number on some chromosome pairs, according to previous reports on reptile groups (Pokorná et al. 2011; Matsubara et al. 2013).

## Conclusions

In this study, the comparison of the cytogenetic maps of two *Cyrtodactylus* species (*C. jarujini* and *C. doisuthep*) enabled us to delineate the process of chromosomal re-organisation in this group. This is the first report in Thailand for the study of cytogenetics of both species. Therefore, the cytogenetic data obtained can be used to benefit cytotaxonomy and the study of evolution of geckos, as well as being an essential prerequisite for future genome projects of gecko groups.
